# Supplementary material for: A ShK-like Domain from Steinernema carpocapsae with Bioinsecticidal Potential
Source: Toxins (Basel). 2022 Nov 2;14(11):754. doi: 10.3390/toxins14110754 (PMC9699480; doi:10.3390/toxins14110754)
Supplement: Supplementary file 1 [file toxins-14-00754-s001.zip › toxins-1952114-supplementary/Supplementary Figures/Supplementary Figure S1.pdf]

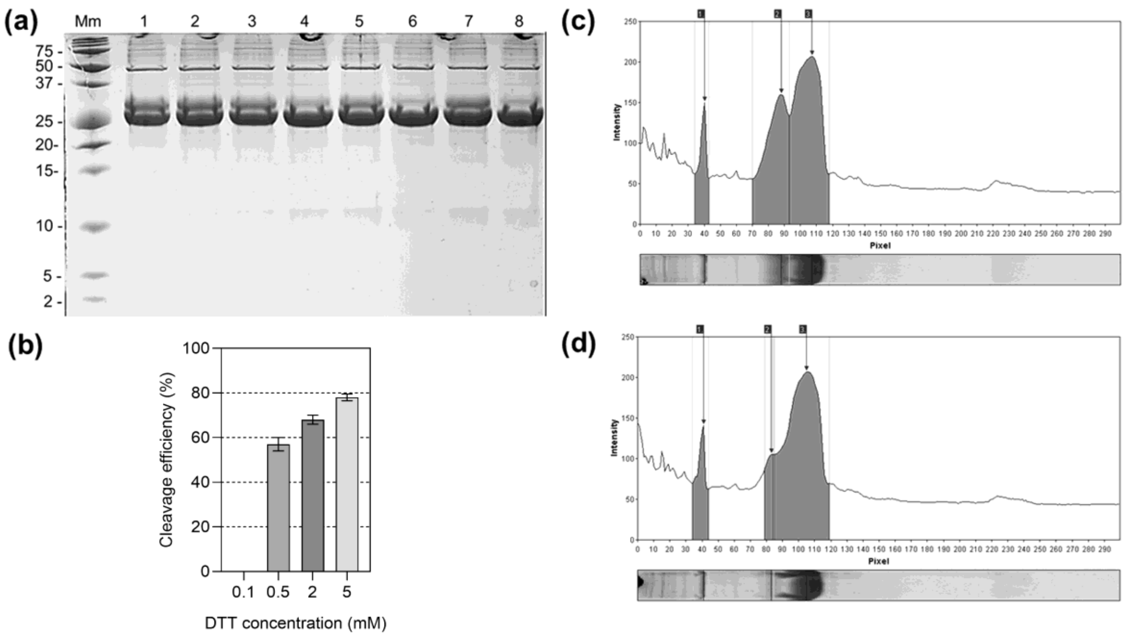

Figure S1: Optimization of DsbC-ScK1 protein cleavage by HRV-3C protease. (**a**) Tris-Tricine SDS-PAGE gel showing cleavage of DsbC-ScK1 fusion protein by HRV-3C protease at a ratio of 1:50 and different concentrations of dithiothreitol (DTT) in 100 mM ammonium acetate buffer, pH 7.0 at 10 °C. Lane Mm: molecular weight protein standards (Mw in kDa); Lanes 1-8, cleavage reactions at increasing DTT concentrations: 0.1 mM (lanes 1 and 2), 0.5 mM (lanes 3 and 4), 2 mM (lanes 5 and 6) and 5 mM (lanes 7 and 8). Lanes 1, 3, 5 and 7, time zero of cleavage; lanes 2, 4, 6 and 8, after 16 h of cleavage. (**b**) Calculated cleavage efficiency of the fusion protein by HRV-3C protease at different DTT concentrations. (**c**) One-dimensional gel electrophoretic peak profile of fusion protein with HRV-3C protease before the start of cleavage reaction. (**d**) One-dimensional gel electrophoretic peak profile of fusion protein with HRV-3C protease after cleavage reaction for 16 hours at optimal DTT concentration. Peak 1, HRV-3C protease; peak 2, DsbC-ScK1; peak 3, DsbC tag; protein peak profiles obtained by Gel Analyzer 19.1 (www.gelanalyzer.com) by Istvan Lazar Jr., PhD and Istvan Lazar Sr., PhD, CSc. The 5 kDa ShK peptide is not visible on the gel at these loaded concentrations
