# Supplementary material for: A ShK-like Domain from Steinernema carpocapsae with Bioinsecticidal Potential
Source: Toxins (Basel). 2022 Nov 2;14(11):754. doi: 10.3390/toxins14110754 (PMC9699480; doi:10.3390/toxins14110754)
Supplement: Supplementary file 1 [file toxins-14-00754-s001.zip › toxins-1952114-supplementary/Supplementary Figures/Supplementary Figure S2.pdf]

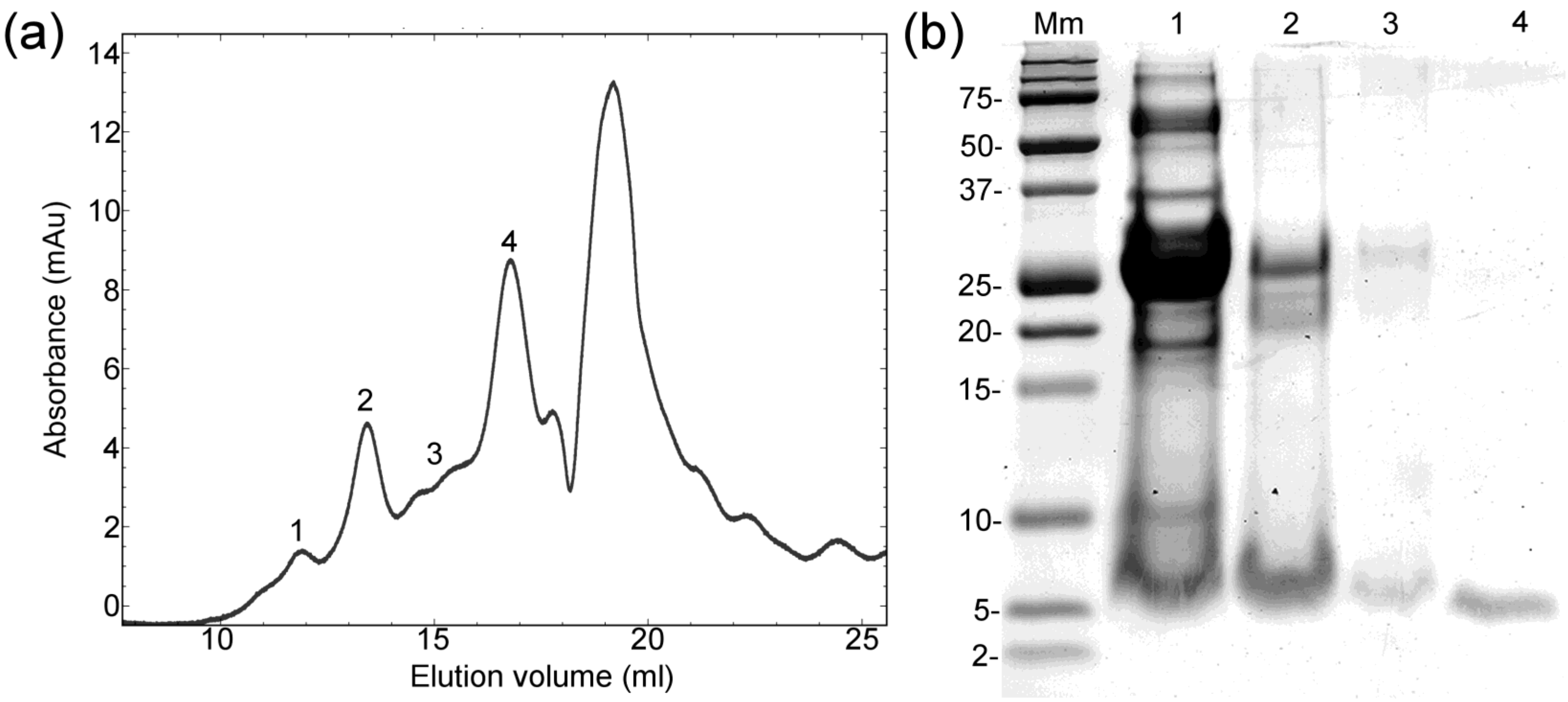

Figure S2: Purification of ScK1 peptide. (**a**) Chromatogram showing the elution of ScK1 peptide during gel filtration chromatography. The ScK1-containing flowthrough from Ni-NTA agarose was applied to an analytical Superdex peptide HR10/300 GL. (**b**) Tris-Tricine SDS-PAGE gel showing protein profile of the different eluted peaks during superdex peptide chromatography. Peak 4 corresponds to the pure target peptide fraction eluted at 16.8 ml of retention volume with an estimated molecular weight of 5 kDa
