# Supplementary material for: A ShK-like Domain from Steinernema carpocapsae with Bioinsecticidal Potential
Source: Toxins (Basel). 2022 Nov 2;14(11):754. doi: 10.3390/toxins14110754 (PMC9699480; doi:10.3390/toxins14110754)
Supplement: Supplementary file 1 [file toxins-14-00754-s001.zip › toxins-1952114-supplementary/Supplementary Figures/Supplementary Figure S3.pdf]

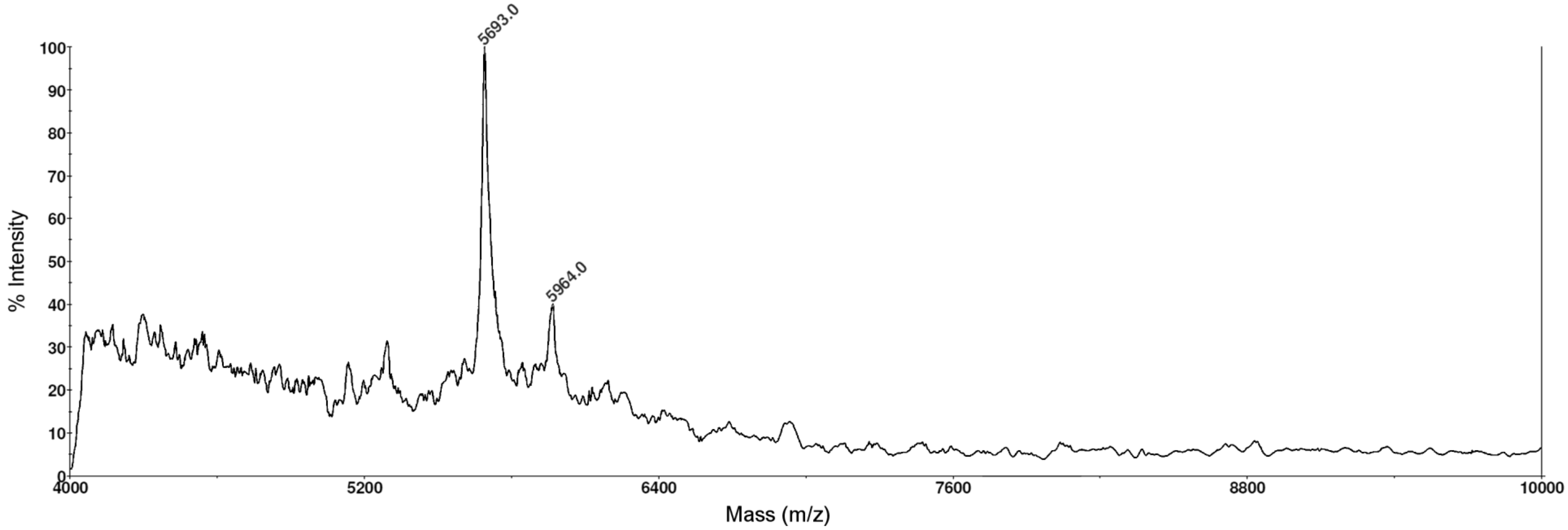

Figure S3: Intact mass measurement of ScK1 peptide by MALDI-TOF/TOF. Linear Mid-Mass Spectrum (m/z range 4000-10000) in positive mode
