# Supplementary material for: A ShK-like Domain from Steinernema carpocapsae with Bioinsecticidal Potential
Source: Toxins (Basel). 2022 Nov 2;14(11):754. doi: 10.3390/toxins14110754 (PMC9699480; doi:10.3390/toxins14110754)
Supplement: Supplementary file 1 [file toxins-14-00754-s001.zip › toxins-1952114-supplementary/Supplementary Figures/Supplementary Figure S4.pdf]

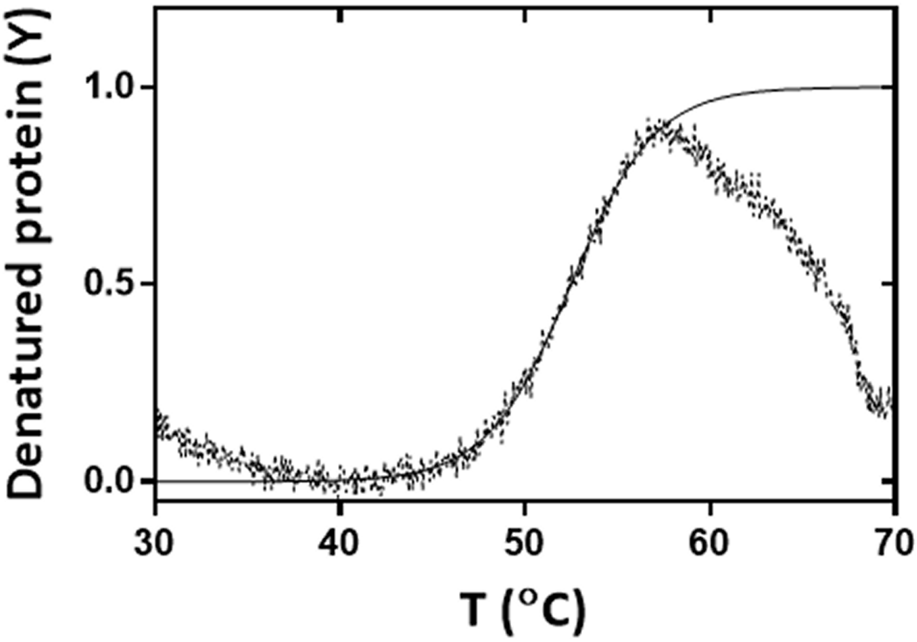

Figure S4: Thermal stability of rScK1. Thermal denaturation profile of rSck1 obtained by differential scanning fluorimetry in the presence of a fluorescent probe that binds to hydrophobic residues (dye DSF). Dye DSF assays were performed in 96-well plates, each well containing (total volume 20 µL) 100 µg·mL-1 ScK1 and 1× Protein Thermal Shift dye. After a 2-min incubation at 25 °C, the temperature was linearly increased from 25 to 90 °C at 1 °C·min-1. Fluorescence emission as a function of temperature was recorded in the ROX channel, data from triplicates were averaged (dotted line) and best fitted with a monophasic sigmoidal function (full line), allowing estimating a melting temperature Tm = 51.8 ± 0.8 °C
